# Intravaginal Administration of Interleukin 12 during Genital Gonococcal Infection in Mice Induces Immunity to Heterologous Strains of *Neisseria gonorrhoeae*

**DOI:** 10.1128/mSphere.00421-17

**Published:** 2018-01-31

**Authors:** Yingru Liu, Julianny Perez, Laura A. Hammer, Heather C. Gallagher, Magdia De Jesus, Nejat K. Egilmez, Michael W. Russell

**Affiliations:** a TherapyX, Inc., Buffalo, New York, USA; b Department of Biomedical Sciences, University at Albany, Albany, New York, USA; c Division of Infectious Diseases, Wadsworth Center, New York State Department of Health, Albany, New York, USA; d Department of Microbiology and Immunology, University of Louisville, Louisville, Kentucky, USA; e Department of Microbiology and Immunology, University at Buffalo, Buffalo, New York, USA; University of Florida

**Keywords:** *Neisseria gonorrhoeae*, adaptive immunity, genital tract immunity, interleukin 12, microencapsulation

## Abstract

It has previously been shown that genital tract infection with *Neisseria gonorrhoeae* in mice does not induce a state of protective immunity against reinfection but instead suppresses the development of adaptive immune responses against *N. gonorrhoeae* dependent on transforming growth factor beta (TGF-β) and interleukin 10 (IL-10). Intravaginal administration during gonococcal infection of IL-12 encapsulated in biodegradable microspheres (IL-12/ms) reverses the immunosuppression and promotes the production of gamma interferon (IFN-γ) and of specific antibodies in serum and genital secretions and accelerates clearance of the infection. In this study, microspheres were shown to remain largely within the genital tract lumen and to release IL-12 over the course of 4 days. Antigonococcal IgA and IgG antibodies induced by IL-12/ms treatment reacted with antigenically different strains of *N. gonorrhoeae* and led to resistance to reinfection with heterologous and homologous strains. Immune resistance to reinfection persisted for at least 6 months after clearance of the primary infection. Experiments performed with immunodeficient strains of mice lacking either IFN-γ or B cells demonstrated that both IFN-γ and B cells were necessary for the IL-12-induced generation of immune responses to *N. gonorrhoeae* and the resulting accelerated clearance of the infection. It is therefore concluded that intravaginally administered IL-12/ms achieves its effect by the sustained release of IL-12 that promotes Th1-driven adaptive immune responses, including the production of specific antigonococcal antibodies that cross-react with multiple strains of *N. gonorrhoeae*. IL-12-enhanced immunity to *N. gonorrhoeae* can be recalled against reinfection after prolonged intervals and is dependent upon both IFN-γ and antibody production by B cells.

**IMPORTANCE** Genital infection with *Neisseria gonorrhoeae* (gonorrhea) is a significant cause of reproductive tract morbidity in women, leading to pelvic inflammatory disease, tubal factor infertility, and increased risk for ectopic pregnancy. WHO estimates that 78 million new infections occur annually worldwide. In the United States, >350,000 cases are reported annually, but the true incidence is probably >800,000 cases/year. Increasing resistance to currently available antibiotics raises concern that gonorrhea might become untreatable. Infection does not induce a state of immune protection against reinfection. Previous studies have shown that *N. gonorrhoeae* suppresses the development of adaptive immune responses by mechanisms dependent on the regulatory cytokines TGF-β and IL-10. This study shows that intravaginal treatment of gonococcal infection in female mice with microencapsulated IL-12 induces persisting anamnestic immunity against reinfection with *N. gonorrhoeae*, even of antigenically diverse strains, dependent on T-cell production of IFN-γ and B-cell production of antibodies.

## Abstract (precis)

Genital infection with *Neisseria gonorrhoeae* (gonorrhea) is a significant cause of reproductive tract morbidity in women, leading to pelvic inflammatory disease, tubal factor infertility, and increased risk for ectopic pregnancy. WHO estimates that 78 million new infections occur annually worldwide. In the United States, >350,000 cases are reported annually, but the true incidence is probably >800,000 cases/year. Increasing resistance to currently available antibiotics raises concern that gonorrhea might become untreatable. Infection does not induce a state of immune protection against reinfection. Previous studies have shown that *N. gonorrhoeae* suppresses the development of adaptive immune responses by mechanisms dependent on the regulatory cytokines TGF-β and IL-10. This study shows that intravaginal treatment of gonococcal infection in female mice with microencapsulated IL-12 induces persisting anamnestic immunity against reinfection with *N. gonorrhoeae*, even of antigenically diverse strains, dependent on T-cell production of IFN-γ and B-cell production of antibodies.

## INTRODUCTION

The sexually transmitted pathogen *Neisseria gonorrhoeae* (the gonococcus) is well-adapted to its natural human host, and it can be acquired repeatedly with little or no evidence of protective immunity arising from prior infections (1, 2). Factors contributing to its success undoubtedly include a remarkable capacity for changing the expression and specificity of most of its major surface antigens (reviewed in reference 3), as well as multiple mechanisms for resisting complement-mediated destruction (4). In addition, we have previously shown that *N. gonorrhoeae* has the capacity to suppress the development of adaptive immune responses governed by T helper (Th) type 1 and 2 cells, while concomitantly inducing Th17-driven innate responses that it appears able to resist (5–7). We have further found that the induced immunosuppression in mice vaginally infected with *N. gonorrhoeae* can be countermanipulated to allow specific immune responses to emerge, with the generation of antigonococcal antibodies and gamma interferon (IFN-γ)-secreting CD4^+^ T cells, the establishment of immune memory, and accelerated clearance of the organism (5–8). Furthermore, when mice are reinfected with *N. gonorrhoeae* 1 month later, the challenge is resisted more effectively than in control mice that had not been treated during the original infection.

The mechanisms of suppression induced by *N. gonorrhoeae* depend on the regulatory cytokines transforming growth factor beta (TGF-β) and interleukin 10 (IL-10) and on the generation of type 1 regulatory T cells (7). Systemic administration of neutralizing antibodies against TGF-β and IL-10 reverses the suppression and allows specific antibody and T-cell responses to develop. We have further found that intravaginal (i.vag.) administration of IL-12 encapsulated in biodegradable microspheres (hereafter designated IL-12/ms) during gonococcal infection similarly promotes the development of Th1-driven immune responses, including production of IFN-γ by CD4^+^ T cells and specific IgG and IgA antibodies in serum and genital secretions, as well as accelerated clearance of the infection, without overtly toxic effects (8). Soluble IL-12 given i.vag. has no effect, and it is far too toxic to be given systemically in effective doses. This raises the question whether encapsulation of IL-12 into polylactic acid (PLA) microspheres works by the sustained delivery of IL-12 over a prolonged period or whether it promotes uptake into the tissues as has been documented for another microparticulate formulation (9). If the latter is correct, then IL-12/ms might release their cytokine load in the vicinity of responsive cells or even be translocated to the draining lymph nodes.

Given the extraordinary antigenic diversity of *N. gonorrhoeae*, it is important to determine whether the immunity against repeat infection induced by treatment with IL-12/ms is limited to the original infecting strain or whether it can extend to challenge with heterologous strains. In addition, the mechanisms of induced immunity to gonococcal infection need to be understood in order to exploit these findings as a potential novel therapeutic modality that would have the further advantage of conferring prophylactic resistance to repeat infection. These considerations assume greater importance with the continuing emergence of antibiotic resistance in this organism, which threatens to render gonorrhea untreatable.

The studies described here were first aimed at tracking the location of fluorescent PLA microspheres in genital tract tissues and iliac lymph nodes (ILN) after vaginal instillation. Release of IL-12 into the vaginal lumen and circulation were also monitored for several days. Second, we determined whether the IL-12-induced immune resistance to reinfection persisted for prolonged periods and whether it extended to challenge with antigenically distinct strains of *N. gonorrhoeae*. Finally, we determined whether enhanced clearance of gonococcal infection due to i.vag. administration depended on the generation of both antibodies and IFN-γ.

## RESULTS

### Microencapsulated IL-12 generates sustained release of IL-12 in the vagina.

**FIG 1:**
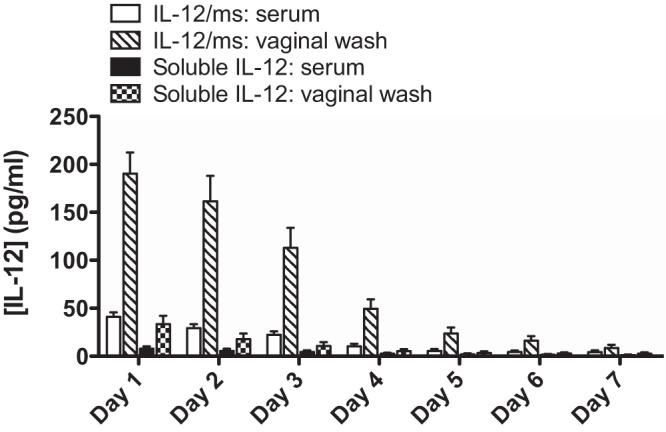
Recovery of IL-12 in serum and vaginal wash fluid samples after i.vag. administration of microencapsulated IL-12 (IL-12/ms) or soluble IL-12. Serum and vaginal wash fluid samples were collected from separate groups of mice on days 1 to 7 after instillation of IL-12/ms or soluble IL-12 (1 µg of IL-12 per mouse) and assayed for IL-12 by ELISA. Values are means (bars) plus standard errors of the means (SEM) (error bars) (*n* = 6 samples).

Female BALB/c mice (six mice in each group) were given one dose of 1.0 µg IL-12 encapsulated in PLA microspheres (IL-12/ms) or in soluble form intravaginally. To avoid removal of the administered dose, serum samples and vaginal wash fluid samples were collected from separate groups of mice on days 1 to 7, and IL-12 was measured by an enzyme-linked immunosorbent assay (ELISA). After a single dose of IL-12/ms, high concentrations of IL-12 were maintained at slowly decreasing levels in vaginal wash fluid samples for up to 4 days (Fig. 1). IL-12 was also detected in the sera of these mice, but the concentrations were low, <40 pg/ml (Fig. 1). The instillation of soluble IL-12 yielded substantially lower recovery of IL-12 in vaginal wash fluid samples, and IL-12 was barely detected in serum (Fig. 1).

**FIG 2:**
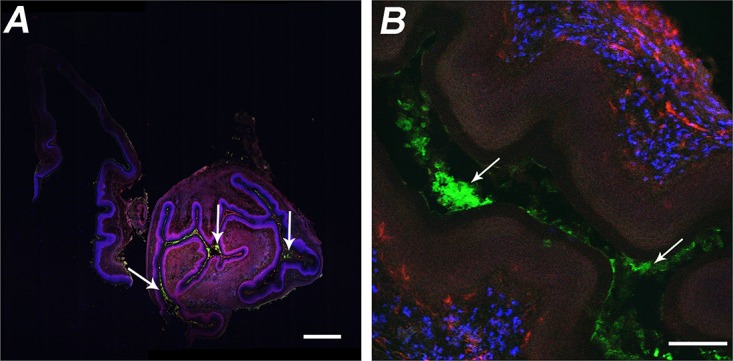
Confocal fluorescence micrographs of vaginal tissue excised from mice 4 h after i.vag. administration of FITC-BSA/ms. (A) FITC-BSA/ms (green) were located in the lumen (white arrows), but no CD4^+^ (red) or CD8^+^ (blue) cells were observed. Bar = 500 µm. (B) Higher magnification showing FITC-BSA/ms (green) located in the lumen (white arrows) and CD11b^+^ (blue) and CD11c^+^ (red) cells in the tissue. Bar = 100 µm.

We further investigated the location of PLA microspheres in genital tract tissues and possibly in the draining ILN after vaginal instillation. A similar dose of PLA microspheres loaded with fluorescein isothiocyanate-labeled bovine serum albumin (FITC-BSA/ms) was instilled intravaginally, and tissues were collected from mice sacrificed 4 or 24 h later. Tissues were also stained for CD11b and CD11c to identify presumptive antigen-presenting cells (dendritic cells and macrophages) or for CD4 and CD8 to identify T cells. Confocal fluorescence microscopy showed that FITC-BSA/ms were largely retained in the lumen of the vagina at 4 h (Fig. 2), and very few were seen within the tissue. CD11b^+^ and CD11c^+^ cells were observed in the tissue (Fig. 2B), but CD4^+^ and CD8^+^ cells were rarely found. No particles were observed in the ILN. FITC-BSA/ms were not reliably detected in tissues obtained at 24 h. These results suggest that IL-12/ms achieve their therapeutic effect by the sustained release of IL-12 at the epithelial surface or within the genital tract tissue, rather than through uptake by antigen-presenting cells and subsequent translocation to the draining lymph nodes.

### Antibodies induced by i.vag. IL-12/ms treatment target both homologous and heterologous strains of *N. gonorrhoeae*.

**FIG 3:**
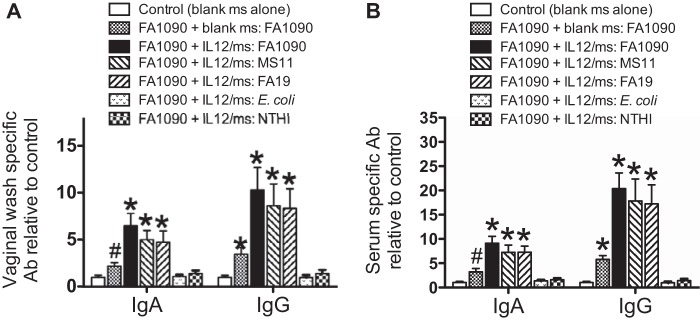
Treatment of mice infected with *N. gonorrhoeae* (FA1090) with IL-12/ms-induced vaginal (A) and serum (B) IgA and IgG antibodies (Ab) detected against homologous (FA1090) and heterologous (MS11 and FA19) strains of *N. gonorrhoeae*, but not against *E. coli* or NTHI. Values are means plus SEM (error bars) (*n* = 5 samples). Values that are significantly different by ANOVA from the values for control samples from uninfected mice treated with blank ms alone are indicated as follows: *, *P* < 0.01; #, *P* < 0.05.

Our previous studies demonstrated that IL-12/ms treatment during gonococcal infection induced the production of vaginal and serum IgG antibodies, as well as vaginal IgA antibodies against the homologous infecting strain of *N. gonorrhoeae* (8). Considering gonococcal surface antigenic variability, we determined whether the induced antibodies could also target heterologous strains of *N. gonorrhoeae*. We therefore measured serum and vaginal wash antibodies by ELISAs using plates with the wells coated with homologous (FA1090) or heterologous (MS11 and FA19) gonococcal strains or with *E. coli* or nontypeable *Haemophilus influenzae* (NTHI). The results showed that vaginal and serum IgA and IgG antibodies to *N. gonorrhoeae* were detected to similar extents against both homologous and heterologous strains (Fig. 3). In contrast, antibodies were not detected above background levels against *E. coli* or NTHI (Fig. 3).

**FIG 4:**
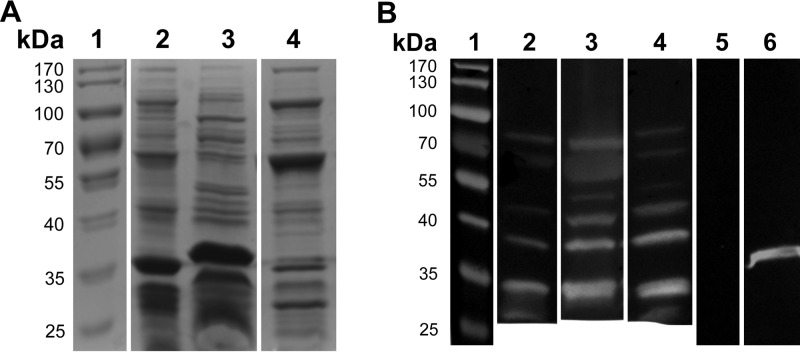
SDS-PAGE of gonococcal OMV preparations and Western blot analysis of serum samples from mice infected with *N. gonorrhoeae* FA1090 against antigens in OMV separated by SDS-PAGE. (A) SDS-polyacrylamide gel stained with Coomassie blue. Lane 1, molecular mass markers (in kilodaltons) (shown to the left of the gel); lane 2, OMV from *N. gonorrhoeae* FA1090; lane 3, OMV from *N. gonorrhoeae* MS11; lane 4, OMV from *N. gonorrhoeae* FA19. (B) Western blots developed for IgG antibodies. Lane 1, molecular mass markers (in kilodaltons) (shown to the left of the gel); lanes 2 to 4, serum from a mouse infected with *N. gonorrhoeae* FA1090 and treated with IL-12/ms, tested against OMV from FA1090 (lane 2), MS11 (lane 3), or FA19 (lane 4); lane 5, serum from a mouse infected with *N. gonorrhoeae* FA1090 and treated with blank ms, tested against OMV from FA1090; lane 6, H5 antibody (anti-porin PIB3) tested against OMV from FA1090.

Antibodies were also analyzed by Western blotting to determine the patterns of specificity for different gonococcal antigens in homologous and heterologous strains, using outer membrane vesicles (OMV) which contain most of the surface antigens expressed by *N. gonorrhoeae*. The protein profiles of *N. gonorrhoeae* FA1090, MS11, and FA19 OMV revealed by sodium dodecyl sulfate-polyacrylamide gel electrophoresis (SDS-PAGE) displayed some quantitative and qualitative differences probably reflecting levels of expression and sequence variation (Fig. 4A). Western blot analyses of serum samples from FA1090-infected mice treated with IL-12/ms against FA1090, MS11, or FA19 OMV separated by SDS-PAGE showed IgG antibodies reactive with bands migrating at ~35 to 80 kDa, with partially similar reactivity against bands present in OMV from all three strains (Fig. 4B). The IgG band at ~35 kDa probably corresponds to porin, as an antiporin antibody (H5) reacted with a band of similar mobility (Fig. 4B). Other bands were visible at approximately 30, 40, 45, 60, and 70 kDa. Further work will be necessary to identify the nature of the antigens detected. However, these results indicated that IL-12/ms treatment of infected mice induced antibodies that recognized antigens in homologous and heterologous strains of *N. gonorrhoeae*.

### Intravaginal IL-12/ms treatment during primary *N. gonorrhoeae* infection induces resistance to reinfection with heterologous strains of *N. gonorrhoeae*.

**FIG 5:**
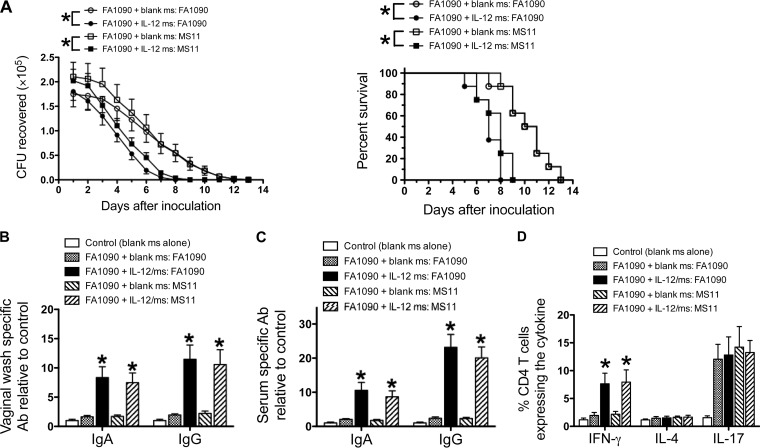
Treatment of mice with IL-12/ms during primary infection with *N. gonorrhoeae* FA1090 induces resistance to reinfection with homologous (FA1090) or heterologous (MS11) strains and induces immune responses to either strain. (A, left) Recovery (mean CFU ± SEM; *n* = 8) of *N. gonorrhoeae* on vaginal swabs after reinfection with strain FA1090 or MS11 as shown. Curves that are significantly different (*P* < 0.01 by ANOVA) are indicated by an asterisk. (Right) Percentage of animals remaining infected at each time point (*P* < 0.001 and <0.005 by Kaplan-Meier analysis), comparing treatment with IL-12/ms versus blank ms, for reinfection with FA1090 and MS11, respectively. (B) Vaginal IgA and IgG antibodies after clearance of secondary infection tested against strain FA1090 or MS11. (C) Serum IgA and IgG antibodies after clearance of secondary infection tested against FA1090 or MS11. (D) Production of IFN-γ, IL-4, and IL-17 by ILN CD4^+^ cells recovered after clearance of secondary infection. In panels B, C, and D, values for mice treated with IL-12/ms that are significantly different (*P* < 0.01 by Student’s *t* test) from the values for mice treated with blank ms are indicated by an asterisk.

We have previously demonstrated that IL-12/ms treatment significantly accelerates the clearance of genital gonococcal infection and induces immune memory and resistance to reinfection with the homologous strain of *N. gonorrhoeae* (8). Given the extraordinary antigenic diversity of *N. gonorrhoeae*, we assessed whether IL-12/ms treatment during primary *N. gonorrhoeae* infection would result in similar resistance to subsequent infection with other strains as reinfection with the same strain. Groups of eight mice infected with *N. gonorrhoeae* FA1090 were treated with IL-12/ms or blank microspheres (blank ms), and after the infection had run its course, the mice were treated with ceftriaxone to ensure complete elimination of the gonococci. One month later, the mice were inoculated with *N. gonorrhoeae* of either strain FA1090 or MS11 without any further treatment, and the course of infection was monitored by vaginal swabbing and plating. The results showed that IL-12/ms treatment during FA1090 infection resulted in similar resistance to reinfection with either FA1090 or MS11 (Fig. 5A). After clearance of the secondary infection, vaginal and serum antibodies were elevated to similar levels against both strains FA1090 and MS11 (Fig. 5B and C). When ILN cells harvested at termination were assayed by flow cytometry for cytokine production by CD4^+^ T cells, and IFN-γ was similarly enhanced in IL-12/ms-treated mice reinfected with either FA1090 or MS11. Consistent with our previous findings, IL-4 production was not enhanced by IL-12/ms treatment during primary infection, and IL-17 production was enhanced in all (re)infected mice regardless of IL-12/ms treatment (Fig. 5D).

In a reciprocal manner, treatment with IL-12/ms during primary infection with *N. gonorrhoeae* MS11 induced resistance to rechallenge with strain FA1090 (see Fig. S1 in the supplemental material).

10.1128/mSphere.00421-17.1 FIG S1  Treatment of mice with IL-12/ms during primary infection with *N. gonorrhoeae* MS11 induces resistance to reinfection with strain FA1090. (Left) Recovery of *N. gonorrhoeae* on vaginal swabs after reinfection with FA1090. *, *P* <0.01 by ANOVA (eight mice in each group). (Right) Percentage of animals remaining infected each day (*P* < 0.002 by Kaplan-Meier analysis) comparing treatment with IL-12/ms versus treatment with blank ms. Download FIG S1, PDF file, 0.5 MB. Copyright © 2018 Liu et al. 2018 Liu et al. This content is distributed under the terms of the Creative Commons Attribution 4.0 International license.

*N. gonorrhoeae* strains FA1090 and MS11 both possess porin of the same major type (PorB.1B). Therefore, to determine whether the major porin type is important to the effect of IL-12/ms treatment on resistance to reinfection, further experiments were performed with strain FA19 (PorB.1A). IL-12/ms treatment during primary infection with strain FA1090 induced resistance to rechallenge with strain FA19 (Fig. S2A). Serum and vaginal IgG and IgA antibody responses assayed after clearance showed cross-reactivity against FA19 (Fig. S2B and C), and IFN-γ CD4 T cells from ILN was elevated (Fig. S2D). Further treatment and reinfection studies using different combinations of *N. gonorrhoeae* strains FA1090, MS11, and FA19 were performed, and similar cross-protections were observed in all cases.

10.1128/mSphere.00421-17.2 FIG S2  Treatment of mice with IL-12/ms during primary infection with *N*. *gonorrhoeae* FA1090 induces resistance to reinfection with homologous (FA1090) or heterologous (FA19) strains and induces immune responses to either strain. (A, left) Recovery of *N*. *gonorrhoeae* on vaginal swabs after reinfection with strain FA1090 or FA19 as shown. *P* < 0.01 (ANOVA) (eight mice in each group). (Right) Percentage of animals remaining infected at each time point. *P* < 0.005 or *P* < 0.0001 comparing treatment with IL-12/ms versus blank ms for reinfection with strain FA1090 or FA19, respectively. (B) Vaginal IgA and IgG antibodies after clearance of secondary infection tested against FA1090 or FA19 as shown. *, *P* < 0.01 (Student’s *t* test) comparing treatment with IL-12/ms versus blank ms (*n* = 5 samples). (C) Serum IgA and IgG antibodies after clearance of secondary infection tested against FA1090 or FA19 as shown. *, *P* <0.01 (Student’s *t* test) comparing treatment with IL-12/ms versus blank ms (*n* = 5 samples). (D) Production of IFN-γ, IL-4, and IL-17 by ILN CD4^+ ^cells recovered after clearance of secondary infection, *, *P* <0.01 (Student’s *t* test) comparing treatment with IL-12/ms versus blank ms (*n* = 5 samples). Download FIG S2, PDF file, 1.9 MB. Copyright © 2018 Liu et al. 2018 Liu et al. This content is distributed under the terms of the Creative Commons Attribution 4.0 International license.

Mice treated with IL-12/ms during primary infection with *N. gonorrhoeae* FA1090 were also resistant to challenge with minimally passaged clinical isolates GC68 (a PorB.1B strain) and GC69 (PorB.1A) (Fig. S3).

10.1128/mSphere.00421-17.3 FIG S3  Treatment of mice with IL-12/ms during primary infection with *N. gonorrhoeae* FA1090 induces resistance to reinfection with minimally passaged clinical isolates GC68 and GC69. (Left) Recovery of *N. gonorrhoeae* on vaginal swabs after reinfection with GC68 or GC69 as shown. *, *P* < 0.01 (ANOVA) (eight mice in each group) comparing treatment with IL-12/ms versus blank ms. (Right) Percentage of animals remaining infected at each time point. *P* <0.0001 or *P* < 0.001 (Kaplan-Meier analysis) comparing treatment with IL-12/ms versus blank ms, for reinfection with GC68 or GC69, respectively. Download FIG S3, PDF file, 1 MB. Copyright © 2018 Liu et al. 2018 Liu et al. This content is distributed under the terms of the Creative Commons Attribution 4.0 International license.

### Duration of the prophylactic effect against reinfection.

**FIG 6:**
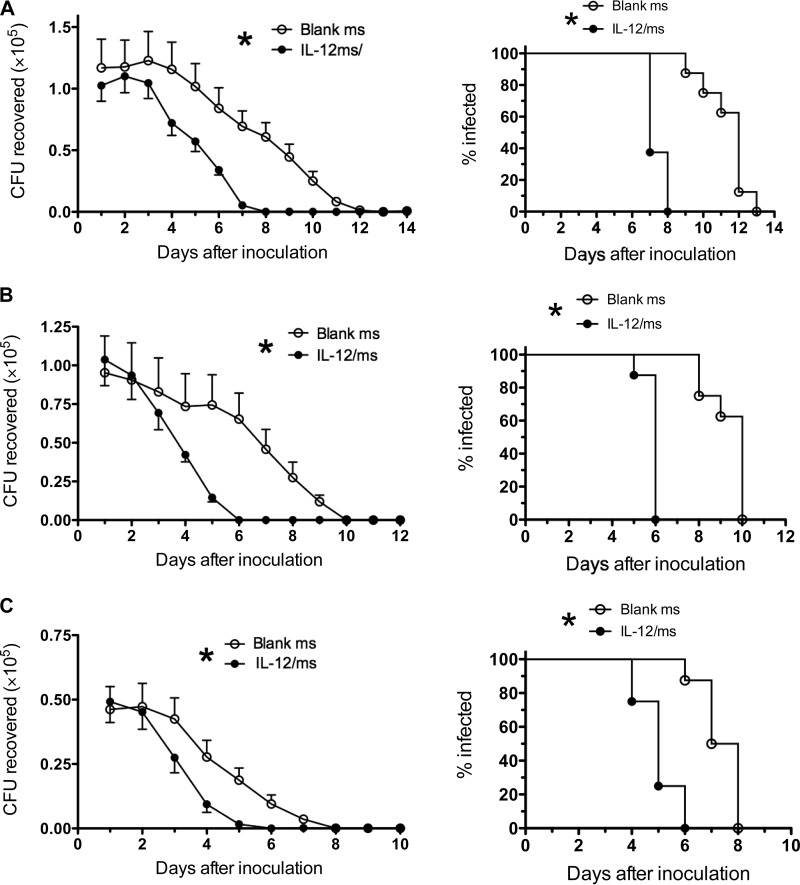
Effect of treatment with IL-12/ms during primary infection with *N. gonorrhoeae* FA1090 against reinfection with *N. gonorrhoeae* MS11 persists for 2 months (A), 4 months (B), or 6 months (C) after the clearance of primary infection. The left panels show recovery (mean CFU plus SEM; *n* = 8) of *N. gonorrhoeae* on vaginal swabs after reinfection with MS11 at each interval. Curves for mice treated with IL-12/ms are significantly different (*P* < 0.01 by ANOVA) from the curves for mice treated with blank ms at each interval (indicated by the asterisk). The right panels show percentage of animals remaining infected at each time point (*P* < 0.001 by Kaplan-Meier analysis), comparing IL-12/ms with blank ms treatment at each interval.

To determine the duration of resistance to reinfection induced by treatment of gonococcal infection with IL-12/ms, groups of mice (eight mice in each group) were infected with *N. gonorrhoeae* FA1090 and treated with IL-12/ms or blank microspheres. After the infection had been cleared (as determined by vaginal swabbing and plating), the mice were treated with ceftriaxone to ensure complete elimination of the gonococci. Two, 4, or 6 months later, the mice were inoculated with *N. gonorrhoeae* MS11 without any further treatment, and the course of infection was monitored by vaginal swabbing and plating. The results showed that the effect of treatment with IL-12/ms persisted for at least 6 months after the primary infection had been cleared (Fig. 6). Although there was a noticeable decline in the clearance times in control mice that had been treated with blank ms during the primary infection (median clearance times of 12 days, 10 days, and 7.5 days after 2, 4, and 6 months, respectively), by comparison, mice that had been treated with IL-12/ms during the primary infection cleared the second infection significantly faster (median clearance times of 7 days, 6 days, and 5 days after 2, 4, and 6 months, respectively) than the corresponding control mice did (Fig. 6).

### Roles of IFN-γ and antibody in accelerated clearance of gonococcal infection due to IL-12/ms treatment.

IL-12/ms treatment of mice infected with *N. gonorrhoeae* induces the production of antibodies specific for gonococcal antigens and the generation of Th1-governed responses as shown by the production of IFN-γ by CD4^+^ T cells. To investigate whether either or both of these aspects of IL-12-enhanced immune responsiveness to *N. gonorrhoeae* are required for the observed accelerated clearance of infection, experiments were performed using mutant C57BL/6 mice deficient in IFN-γ (IFN-γ-KO [KO stands for knocked out]) or B cells (μMT). Groups of wild-type C57BL/6 (control) mice and immunodeficient mice (eight mice in each group) were infected with *N. gonorrhoeae* FA1090 and treated with IL-12/ms or blank ms as described above. The course of vaginal gonococcal infection was not altered in control (blank-ms-treated) immunodeficient mice relative to wild-type mice. All wild-type and immunodeficient mice started to reduce the recoverable gonococcal load from day 5 or 6 onwards and had cleared the infection by days 9 to 16 (median, 10 to 15 days).

**FIG 7:**
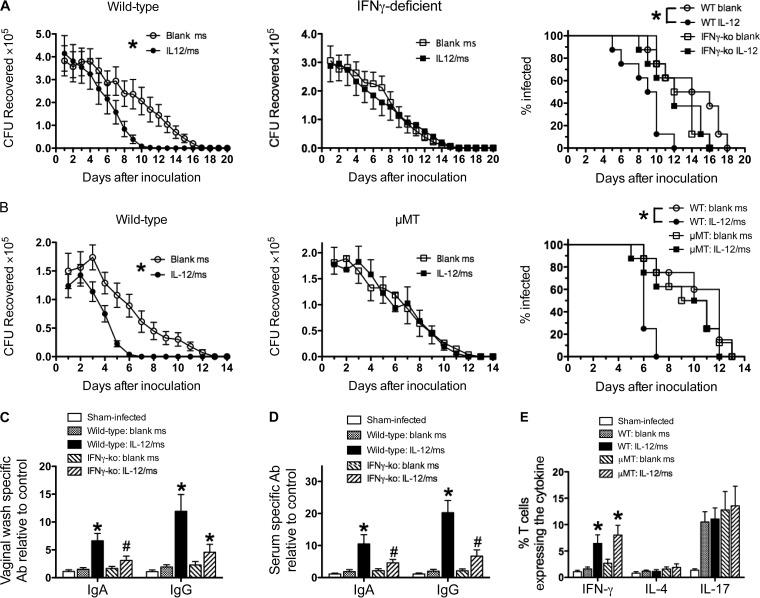
Therapeutic effect of IL-12/ms treatment on gonococcal infection depends on both IFN-γ and B cells. (A) Accelerated clearance of *N. gonorrhoeae* by treatment with IL-12/ms is abrogated in IFN-γ-deficient (IFN-γ-KO) mice compared to wild-type mice. (Left and middle) Recovery (mean CFU ± SEM; *n* = 8) of *N. gonorrhoeae* on vaginal swabs after infection with strain FA1090 in wild-type (left) and IFN-γ-KO (middle) mice. Curves are significantly different (*P* < 0.01 by ANOVA) comparing treatment with IL-12/ms versus blank ms for wild-type (WT) mice. Curves are not significantly different (NS) for IFN-γ-KO mice. (Right) Percentage of mice remaining infected at each time point. Curves are significantly different (*P* < 0.005 by Kaplan-Meier analysis) comparing treatment with IL-12/ms versus blank ms for wild-type mice. Curves are not significantly different (NS) for IFN-γ-KO mice. (B) Accelerated clearance of *N. gonorrhoeae* by treatment with IL-12/ms is abrogated in B-cell-deficient (µMT) mice compared to wild-type mice. (Left and middle) Recovery (mean CFU ± SEM; *n* = 8) of *N. gonorrhoeae* on vaginal swabs after infection with FA1090 in wild-type (left) and µMT (middle) mice. Curves are significantly different (*P* < 0.01 by ANOVA) comparing treatment with IL-12/ms versus blank ms for wild-type mice. Curves are not significantly different (NS) for µMT mice. (Right) Percentage of mice remaining infected at each time point. Curves are significantly different (*P* < 0.002 by Kaplan-Meier analysis) comparing treatment with IL-12/ms versus blank ms for wild-type mice. Curves are not significantly different (NS) for µMT mice. (C and D) Vaginal (C) and serum (D) antibody responses induced by IL-12/ms treatment are diminished in IFN-γ-deficient (IFN-γ-KO) compared to wild-type mice. Values that are significantly different (by Student’s *t* test) for mice treated with IL-12/ms from values for mice treated with blank ms are indicated as follows: *, *P* < 0.01; #, *P* < 0.05. (E) IFN-γ responses induced by treatment with IL-12/ms are maintained in B-cell-deficient (µMT) mice. Values that are significantly different (*P* < 0.01 by Student’s *t* test) comparing treatment with IL-12/ms versus blank ms in both wild-type and µMT mice are indicated by an asterisk.

In contrast to wild-type mice, clearance of gonococcal infection was not accelerated in IFN-γ-KO mice treated with IL-12/ms compared to mice treated with blank ms (Fig. 7A). Likewise, µMT mice treated with IL-12/ms did not show accelerated clearance of gonococcal infection (Fig. 7B). Thus, deficiency of either IFN-γ or B cells abrogated the therapeutic effect of IL-12/ms in accelerating clearance of genital gonococcal infection. The production of gonococcus-specific vaginal and serum IgA and IgG antibodies induced by IL-12/ms treatment in wild-type mice was diminished in IFN-γ-KO mice (Fig. 7C and D), and as expected, there was no generation of IFN-γ by the ILN T cells from IL-12/ms-treated IFN-γ-KO mice (not shown). Similarly, no antibody response to IL-12/ms treatment during infection was detected in μMT mice (not shown). In contrast, the enhanced production of IFN-γ by CD4^+^ T cells isolated from the ILN of μMT mice treated with IL-12/ms was not affected (Fig. 7E). Furthermore, as noted previously, there was no IL-4 response, and IL-17 responses induced by the gonococcal infection itself remained unaltered (Fig. 7E). These findings indicate that accelerated clearance induced by IL-12/ms treatment in mouse *N. gonorrhoeae* infection depended on both IFN-γ and antibody production by B cells.

## DISCUSSION

We have previously shown that the adaptive immune response to genital gonococcal infection in mice is suppressed by mechanisms that involve the regulatory cytokines TGF-β and IL-10 and the generation of type 1 regulatory T cells (5–7) but that this immunosuppression can be reversed by administering neutralizing antibodies to TGF-β and IL-10 (6, 7) or by the i.vag. delivery of microencapsulated IL-12 (8). IL-12/ms treatment of mice infected with *N. gonorrhoeae* allows the emergence of Th1-driven specific immune responses, including the production of antigonococcal antibodies, secretion of IFN-γ by CD4^+^ T cells, establishment of recallable immune memory, accelerated clearance of the existing infection, and resistance to reinfection by the same strain of *N. gonorrhoeae* 1 month later (8). The experiments reported here demonstrate that the effect of i.vag.-administered IL-12/ms on immunity to gonococcal infection persisted for at least 6 months, that it extended to antigenically different, heterologous strains of *N. gonorrhoeae*, and that it depended upon both IFN-γ and B cells.

IL-12 is a key cytokine for driving Th1-dependent adaptive immune responses (10), and it counteracts the suppressive effects of regulatory T cells (Tregs) and cytokines TGF-β and IL-10 (11). Indeed, IL-12 was proposed for the treatment of certain cancers in which Tregs and TGF-β play a role in suppressing the activity of tumor-associated CD4^+^ and CD8^+^ T cells, but the systemic administration of soluble IL-12 in effective doses is dangerously toxic (12). To avoid this problem, microencapsulation of IL-12 in biodegradable microspheres was developed to deliver sustained but low therapeutic concentrations of the cytokine in the vicinity of tumors (13). We found that this formulation of IL-12 delivered i.vag. in *N. gonorrhoeae*-infected mice was effective in reversing the gonococcus-induced immunosuppression (8). IL-12 was released into the vaginal lumen after i.vag. instillation of IL-12/ms over the course of ~4 days, consistent with the *in vitro* release of IL-12 from IL-12/ms suspended in phosphate-buffered saline (PBS) over the same time period (unpublished observations). In contrast, the same dose of soluble IL-12 delivered i.vag. dissipated rapidly, and we previously showed that it had no measurable effect on the clearance of gonococcal infection or the development of an immune response (8). Only low concentrations of IL-12 (<40 pg/ml) were detected in the circulation system after i.vag. administration of IL-12/ms, and no overt ill effects were observed in mice treated with this dose (1 µg) of IL-12/ms, which we previously showed to be sufficient for the desired therapeutic effect in dose-response experiments (8). It has been reported that protein-coated nanoparticles (20 to 40 nm) can be taken up by epithelial cells in the female genital tract and induce immune responses to the coupled protein (9). However, we found that our preparations of polylactic acid microspheres (0.1 to 5 µm) remained largely within the genital tract lumen. Thus, it appears that our formulation of IL-12/ms achieves its effect by the sustained release of IL-12 at the mucosal surface and tissues of the genital tract, rather than by translocation to its draining lymph nodes.

Immune resistance to reinfection induced by IL-12/ms treatment during primary infection persisted for at least 6 months. It is not practically possible to extend the interval before reinfection for longer than 6 months, as mice become increasingly resistant to gonococcal infection as they age. This effect can be seen in Fig. 6 where the control mice treated with blank ms during primary infection cleared the challenge reinfection faster as the interval increased from 2 months to 4 months to 6 months. Nevertheless, the effect of prior IL-12/ms treatment in accelerating clearance of the secondary infection was still seen after each interval. Assay of the antibodies present in serum and vaginal wash fluid samples after clearance of the secondary infection at 6 months indicated that recallable immune memory was established, because we have noted that antibody levels tend to decline over several months in the absence of restimulation. IL-12-induced IFN-γ expression by CD4^+^ T cells in ILN also declines over 2 to 4 months after primary infection, but the finding that ILN CD4^+^ T cells collected from mice reinfected 6 months after the primary infection expressed IFN-γ corroborates the establishment and recall of memory within the Th1 cell population.

The importance of both antibodies and IFN-γ for the observed development of immune resistance to gonococcal infection induced by treatment with IL-12/ms was demonstrated by the use of immunodeficient mice lacking either B cells (µMT) or IFN-γ. The effect of treatment of gonococcal infection with IL-12/ms was abrogated in both of these mutants. IFN-γ production was maintained in µMT mice, but this was inadequate to accelerate clearance; thus, we infer that antibody production by B cells is critical for immune clearance of *N. gonorrhoeae*. However, IFN-γ-deficient mice also had diminished antibody responses despite IL-12 treatment during the infection. This suggests that IFN-γ is required for antibody responses to *N. gonorrhoeae*, but it is also possible that IFN-γ contributes in other ways to defense against *N. gonorrhoeae*. For example, it is known that IFN-γ activates phagocytes, including both neutrophils and macrophages, and upregulates the expression of receptors for IgG, especially the high-affinity Fc gamma receptor I (FcγR-I) (14, 15). Whether other IFN-γ-induced cell-mediated immune mechanisms, such as cytotoxic activity, can contribute to immune defense against *N. gonorrhoeae* seems unlikely, as it is largely an extracellular infection, but this possibility cannot be formally ruled out. Although Th2-driven immune responses are important for certain types of antibody formation, notably the IgG1 subclass in mice, production of the Th2-driving cytokine IL-4 was not seen and moreover was not expected for responses induced by IL-12, which drives Th1 responses. In contrast, IFN-γ drives the development in mice of IgG2a antibodies, which are the most effective isotype of murine IgG in complement activation (16) and in opsonization (17). Thus, it is plausible to hypothesize that IL-12 treatment of gonococcal infection leads to the generation of antibodies that would be most effective in promoting complement-mediated and phagocytic defense against *N. gonorrhoeae*. However, other mechanisms cannot be ruled out. While IgG occurs in murine genital secretions, its concentration is about 10-fold lower than that of IgA in vaginal wash fluid samples (18). Both IgA and IgG antibodies were elevated in vaginal wash fluid samples by treatment of the infection with IL-12/ms, but the relative proportions of these two classes of specific antigonococcal antibodies cannot be determined from the assays used without appropriate calibration standards, which are not available.

*N. gonorrhoeae* is well-known for its extraordinary antigenic variability, involving the expression and composition of many of its known surface antigens, including porin (19), lipo-oligosaccharide (20), opacity (Opa) proteins (21), and pilus protein (22). All of these proteins as well as other proteins have been proposed as vaccine antigens (23). However, none has yet been developed as a practicable vaccine. It was therefore surprising to find that immunity to *N. gonorrhoeae* induced by the treatment of infection with IL-12/ms resulted in prophylactic resistance to subsequent reinfection, not only with the same strain as used in the primary infection but also against other heterologous strains known to express different antigens. The full extent of this cross-protection is not known at present, as it would require considerable effort to determine this experimentally even with relatively few isolates from disparate sources. However, we note that the cross-protective effect appears to be independent of the major porin type (PorB.1A versus PorB.1B), and likely also of Opa protein type, as these protein types differ between the strains tested (24–26). In addition, we observed cross-protection against reinfection with minimally passaged clinical isolates as well as between the widely used but still virulent “laboratory” strains FA1090, MS11, and FA19. Initial examination of antigens recognized by antibodies in sera from IL-12/ms-treated, infected mice reveals several cross-reactive antigens present in the different strains. Identification of the important antigens involved in cross-protective immunity will require extensive further studies using proteomic technology, with serum and vaginal wash fluid samples from multiple animals as well as numerous gonococcal isolates.

It is well-known that human gonorrhea can be acquired repeatedly, with apparently no generation of protective immunity to reinfection, or even of measurable immune responses that reliably correlate with infection or predict resistance to reinfection (1, 3). It has therefore been impossible to define a state of protective immunity against gonorrhea in humans, and consequently, the determinants or even correlates of immune protection against *N. gonorrhoeae* in humans remain unknown. This situation is also reflected in mice, which are the only available species in which genital gonococcal infection has been established for the study of immune responses to this otherwise exclusively human infection (27–31). Thus, while *N. gonorrhoeae* is eliminated typically within 2 to 3 weeks by mechanisms that remain uncertain but may simply reflect the fact that *N. gonorrhoeae* is not adapted to survive in mice, there is no measurable antibody response, and animals that have recovered from the infection can be reinfected even by the same strain with the same profile of clearance, indicating no inducible protective immunity arising from the prior infection. We have repeatedly found that genital gonococcal infection in mice induces the generation of Th17 responses as shown by the production of IL-17 by CD4^+^ T cells, regardless of any treatment (8, 30; this study). IL-17 (and its “sister” Th17 cytokine, IL-22) are known to elicit innate defense mechanisms, including the secretion of antimicrobial proteins by epithelial cells, and the recruitment of phagocytes, especially neutrophils, which are the characteristic feature of the purulent discharge associated with clinical gonorrhea. However, there is accumulating evidence that *N. gonorrhoeae* can survive within neutrophils and resist antimicrobial proteins such as defensins (32, 33). Thus, we have hypothesized that *N. gonorrhoeae* proactively induces Th17-driven innate responses that it can at least partially resist and that it concomitantly suppresses Th1- and Th2-driven adaptive immune responses that would eliminate it (31). Our findings demonstrate that treatment with IL-12/ms reverses the suppression of adaptive immunity by inducing Th1-driven responses, including the production of IFN-γ and specific antigonococcal antibodies, without compromising Th17 responses. This affords a new approach to therapeutic treatment of gonorrhea that has the additional advantage of inducing prophylactic resistance to reinfection.

It has recently been reported that subjects in New Zealand who received the meningococcal outer membrane vesicle vaccine (MeNZB) showed reduced risk of infection with gonorrhea over the ensuing few years (34). Our studies of gonococcal immunity in mice parallel these important findings in revealing that a state of immune protection against *N. gonorrhoeae* can be developed. The finding that therapeutic treatment of gonococcal infection with IL-12/ms has prophylactic potential against reinfection implies that IL-12/ms serves as an immunomodulatory adjuvant and in effect turns the existing, nonimmunogenic infection into a live vaccine. We have exploited this observation to construct an experimental nonliving vaccine consisting of gonococcal OMV plus IL-12/ms administered i.vag. (35). Both modalities of IL-12/ms intervention generate essentially similar responses in terms of Th1-driven IFN-γ and antibody production, establishment of immune memory, and immune defense against gonococcal infection (35; this study). As such, results of the two studies are mutually supportive, and moreover, they afford the means to investigate in further detail the mechanisms responsible for immune defense against *N. gonorrhoeae*.

## MATERIALS AND METHODS

### Mice.

Female wild-type BALB/c mice were purchased from Jackson Laboratories (Bar Harbor, ME) and used for the experiments unless otherwise specified. Female wild-type C57BL/6 mice and immunodeficient strains B6.129S7-*Ifng*^*tm1Ts*^/J (gamma interferon [IFN-γ] deficient), B6.129S2-*Ighm*^*tm1Cgn*^/J (B cell deficient; also known as µMT) were also obtained from Jackson Laboratories. Mice were maintained in a biosafety level 2 (BSL2) suite in the Laboratory Animal Facility at the University at Buffalo, which is fully accredited by AAALAC. All animal protocols were approved by the Institutional Animal Care and Use Committee of the University at Buffalo.

### Bacteria.

*Neisseria gonorrhoeae* strain FA1090 was provided by Ann Jerse (Uniformed Services University of the Health Sciences, Bethesda, MD), strain MS11 was provided by Daniel Stein (University of Maryland), and strains FA19, GC68, and GC69 were provided by Marcia Hobbs (University of North Carolina at Chapel Hill). Strains GC68 and GC69 were derived from minimally passaged clinical isolates 9087 and 0336, respectively, and transformed with the streptomycin resistance *rpsL* gene (35). *Escherichia  coli* K-12 was provided by Terry Connell (University at Buffalo). Nontypeable *Haemophilus influenzae* (NTHI) strain 6P24H1 was provided by Timothy Murphy (University at Buffalo). *N. gonorrhoeae* was cultured on GC agar supplemented with hemoglobin and IsoVitaleX (BD Diagnostic Systems, Franklin Lakes, NJ) at 37°C in air with 5% CO_2_, and the resultant growth was checked for colony morphology consistent with Opa protein and pilus expression. NTHI was cultured on GC agar supplemented with hemoglobin only. *E. coli* was cultured on brain heart infusion (BHI) agar. Bacterial cell density was determined by measuring optical density at 600 nm and referring to a previously determined calibration curve.

### IL-12 microspheres.

Murine interleukin 12 (IL-12) (Wyeth, Philadelphia, PA) was encapsulated into polylactic acid microspheres (IL-12/ms) using the phase inversion nanoencapsulation (PIN) technology as previously described (36) except that bovine serum albumin (BSA) was replaced by sucrose (0.1%, wt/wt). Control (blank) microspheres (blank ms) were prepared in the same way but without IL-12.

For studies on tissue uptake, PIN microspheres were prepared using bovine serum albumin labeled with fluorescein isothiocyanate (FITC).

### Histology for uptake of microspheres.

Vaginal tissue and iliac lymph nodes (ILN) were harvested from euthanized mice 4 or 24 h after intravaginal (i.vag.) instillation of microspheres loaded with FITC-labeled bovine serum albumin (FITC-BSA/ms), embedded in Tissue-Plus optimal cutting temperature (OCT) compound (Fisher Health Care, Houston, TX, USA), and snap-frozen in liquid nitrogen. Serial cryosections (25 μm) were prepared at −21°C with a Cryostar NX70 cryostat (Thermo Scientific, Kalamazoo, MI). Cryosections were kept at room temperature for at least 24 h prior to staining. Fluorochrome-conjugated antibodies were applied sequentially (37, 38) in the following order: T cells were first stained with CD4 antibody labeled with phycoerythrin (PE) (eBioscience, San Diego, CA), and CD8α labeled with Alexa Fluor 647 (BD Pharmingen), followed by the macrophage marker CD11b labeled with Alexa Fluor 633 (eBioscience) or the dendritic cell (DC) marker CD11c labeled with Alexa Fluor 633 (BD Pharmingen). Antibodies were diluted with phosphate-buffered saline (PBS) (pH 7.4) containing 2% fetal calf serum; antibodies were diluted 1:5 for CD4 and CD8α and 1:10 for CD11b and CD11c and incubated at 37°C for 40 min. Images were captured using a Leica SP5 confocal laser scanning microscope (Leica, Wetzlar, Germany) and processed using Fiji software (39). Panels containing confocal images were generated using Adobe Photoshop version 13.0 x32. The images were uniformly brightened by 20% for clarity and marked using the drawing tools to highlight the results and to provide orientation of the tissues. No other image manipulations were performed.

### Vaginal infection and treatment schedule.

Female mice between 7 and 9 weeks old were prepared for infection by subcutaneous (s.c.) injection on days −2, 0, and 2 of 0.5 mg Premarin (Pfizer, Philadelphia, PA), commenced on an antibiotic regime of vancomycin and streptomycin injected s.c., plus trimethoprim sulfate in the drinking water, and infected i.vag. on day 0 by instillation of 5 × 10^6^ CFU of freshly grown *N. gonorrhoeae* strain FA1090 as described previously (8, 27). Mice were then treated i.vag. with IL-12/ms (containing 1 µg IL-12) in 40 µl PBS given on day 0 (approximately 6 h after infection) and days 2 and 4 (8). Control groups were treated with blank ms alone.

After the primary infection had run its course and been cleared, as determined by vaginal swabbing and plating (see below), mice were treated with ceftriaxone (300 µg, intraperitoneal) to ensure complete elimination of the infection. One to six months later, mice were prepared for reinfection by treatment with Premarin and antibiotics and challenged i.vag. with 5 × 10^6^ CFU of *N. gonorrhoeae* FA1090, MS11, FA19, GC68, or GC69 as described in Results (8). Vaginal swabs were collected daily, quantitatively diluted, and plated on GC agar supplemented with hemoglobin, IsoVitaleX, and selective growth inhibitors (vancomycin, streptomycin, nisin, colistin, and trimethoprim). The limit of detection was 100 CFU recovered per mouse. An individual who was blinded to experimental treatments counted gonococcal recovery, and all experiments were repeated two or three times for verification. Results shown are from one experiment. As reported previously (8, 35), a high degree of reproducibility was obtained in replicate experiments, and data from replicate experiments are available on request.

### Assay of serum and vaginal antibodies.

Vaginal wash fluid and serum samples were collected from mice at the indicated time points. IgA and IgG antibodies were measured by an enzyme-linked immunosorbent assay (ELISA) of plates containing wells coated with whole gonococci and using alkaline phosphatase-conjugated goat anti-mouse IgA or IgG reagents (Southern Biotech, Birmingham, AL) and *p*-nitrophenyl phosphate substrate as described previously (5). H5 mouse monoclonal antibody (specific for *N. gonorrhoeae* porin serovar PIB3; obtained from Marcia Hobbs) was used to establish standard curves for IgG antibody assays. The total IgA and IgG concentrations in vaginal wash fluid samples were assayed by ELISA of wells coated with anti-IgA or anti-IgG (Southern Biotech). Antibody data were expressed relative (fold increase) to the antibody levels detected in control samples (from sham-infected mice) assayed simultaneously.

### Cytokine production.

Cells isolated from ILN were assayed for cytokine production by intracellular staining and flow cytometry as described previously (8), using antibodies to mouse CD4, IFN-γ, IL-4, and IL-17A conjugated with FITC, PE, or allophycocyanin (eBioscience).

### Sodium dodecyl sulfate-polyacrylamide gel electrophoresis (SDS-PAGE) and Western blotting.

Gonococcal outer membrane vesicles (OMV) were prepared by shearing in lithium acetate buffer as described previously (35). OMV preparations were boiled for 5 min in SDS loading buffer containing 2-mercaptoethanol. Protein quantification was done with the RC DC protein assay kit (Bio-Rad, Hercules, CA). From each sample, 10 μg of protein was electrophoresed on 10% Mini-PROTEAN TGX precast gels (Bio-Rad). A prestained protein ladder (PageRuler; Thermo Fisher Scientific) was used as a molecular mass marker. Replicate gels were either stained with Coomassie blue or transferred to nitrocellulose membranes using the electrophoresis transfer system (Bio-Rad). Membranes were blocked in PBS containing 3% skim milk overnight at 4°C and incubated for 2 h with serum samples diluted 1:200 in PBS plus 3% skim milk. Specific antibodies bound to *N. gonorrhoeae* OMV preparations were detected with horseradish peroxidase-conjugated goat anti-mouse IgG or anti-mouse IgA (Santa Cruz Biotechnology, Paso Robles, CA) diluted 1:4,000 in PBS plus 3% skim milk. The Pierce (Rockford, IL) detection kit was used for chemiluminescence detection, and images were collected with a ChemiDoc MP imaging system (Bio-Rad).

### Statistical analysis.

Data on recovery of *N. gonorrhoeae* (CFU) on vaginal swabs were analyzed by two-way analysis of variance (ANOVA) for repeated measures with Fisher’s protected least significant difference test. Clearance of infection, defined as the first of 3 consecutive days of zero recovery of *N. gonorrhoeae*, was analyzed by Kaplan-Meier analysis with log rank test. Immune response data were analyzed by Student’s *t* (unpaired, two-tailed) for comparing two groups or by ANOVA with Bonferroni test for multiple comparisons. A *P* value of <0.05 was considered statistically significant. Data were compiled and analyzed using Microsoft Excel and Prism 5 (GraphPad Software, San Diego, CA).
