# Supplementary figures and images for: Intravaginal Administration of Interleukin 12 during Genital Gonococcal Infection in Mice Induces Immunity to Heterologous Strains of Neisseria gonorrhoeae
Source: mSphere. 2018 Jan 31;3(1):e00421-17. doi: 10.1128/mSphere.00421-17 (PMC5793040; doi:10.1128/mSphere.00421-17)

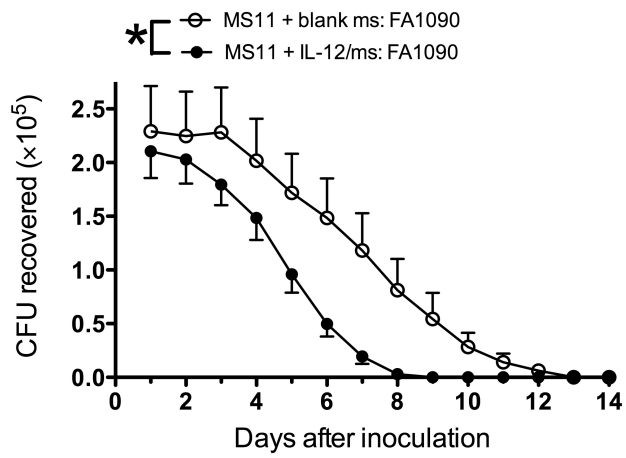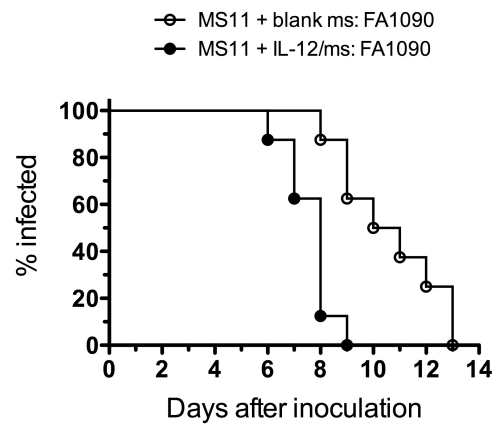

Supplement: FIG S1 [file sph001182467sf1.pdf]

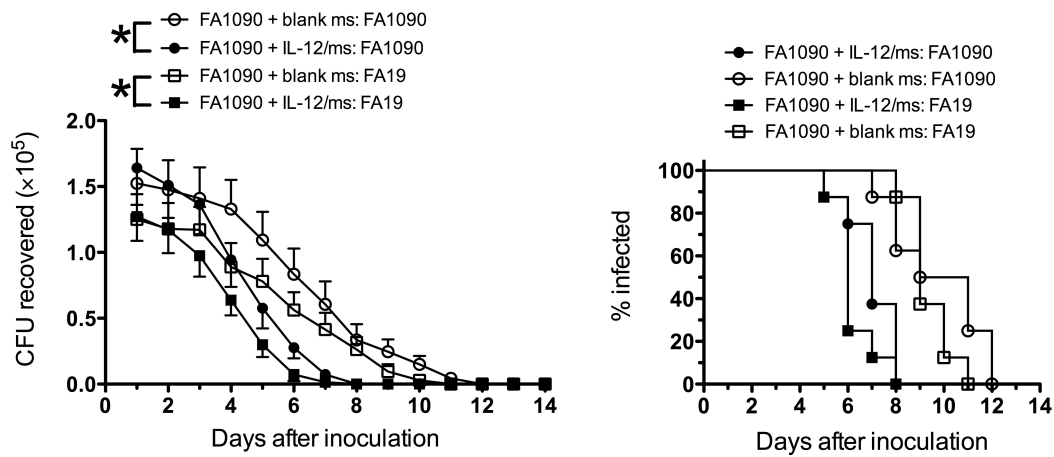

**A**

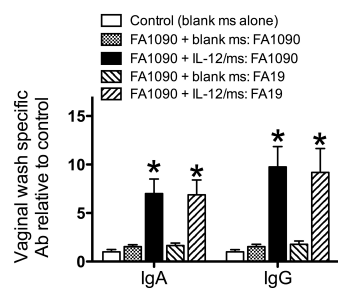

**B**

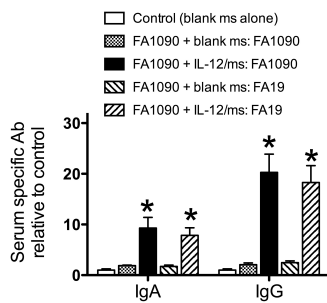

**C**

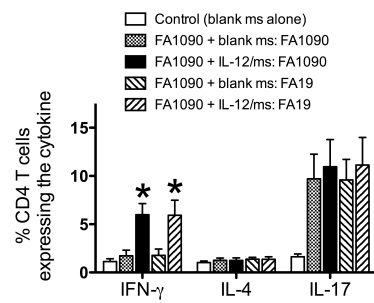

**D**

Supplement: FIG S2 [file sph001182467sf2.pdf]

### Reinfection with GC68

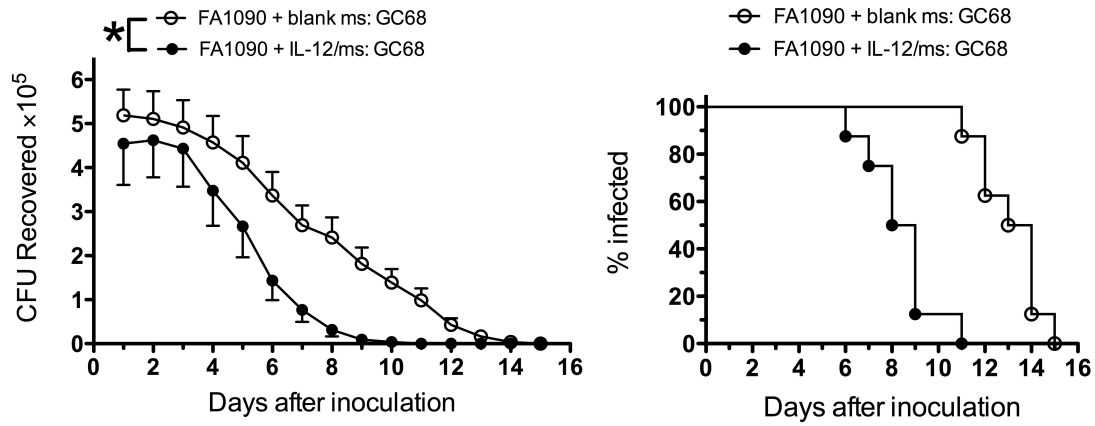

### Reinfection with GC69

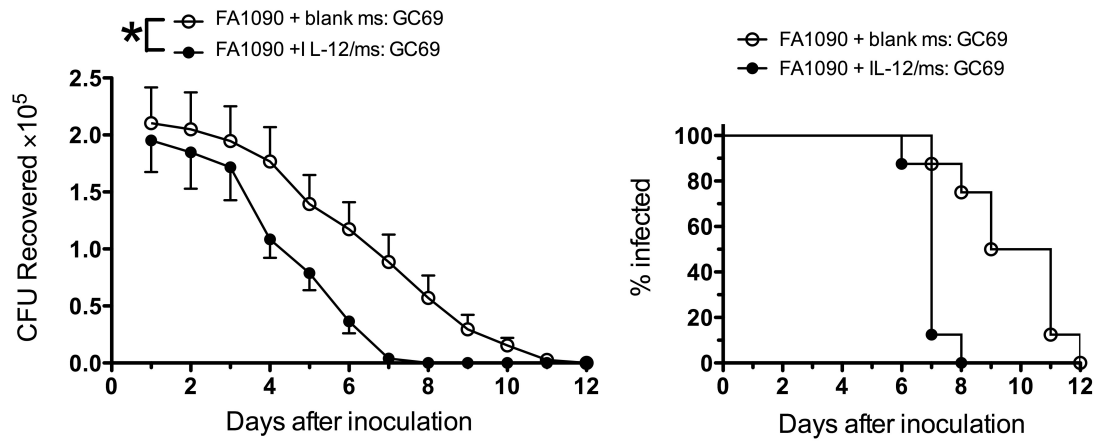

Supplement: FIG S3 [file sph001182467sf3.pdf]
